# A comprehensive review on efficient artificial intelligence models for classification of abnormal cardiac rhythms using electrocardiograms

**DOI:** 10.1016/j.heliyon.2024.e26787

**Published:** 2024-02-29

**Authors:** Utkarsh Gupta, Naveen Paluru, Deepankar Nankani, Kanchan Kulkarni, Navchetan Awasthi

**Affiliations:** a Department of Computational and Data Sciences, Indian Institute of Science, Bengaluru, 560012, India; b Department of Computer Science and Engineering, Indian Institute of Technology, Guwahati, Assam, 781039, India; c IHU-LIRYC, Heart Rhythm Disease Institute, Fondation Bordeaux Université, Pessac, Bordeaux, F-33000, France; d University of Bordeaux, INSERM, Centre de recherche Cardio-Thoracique de Bordeaux, U1045, Bordeaux, F-33000, France; e Faculty of Science, Mathematics and Computer Science, Informatics Institute, University of Amsterdam, Amsterdam, 1090 GH, the Netherlands; f Department of Biomedical Engineering and Physics, Amsterdam UMC, Amsterdam, 1081 HV, the Netherlands

**Keywords:** Artificial intelligence, Deep learning, Lightweight models, ECG classification, Arrhythmia's

## Abstract

Deep learning has made many advances in data classification using electrocardiogram (ECG) waveforms. Over the past decade, data science research has focused on developing artificial intelligence (AI) based models that can analyze ECG waveforms to identify and classify abnormal cardiac rhythms accurately. However, the primary drawback of the current AI models is that most of these models are heavy, computationally intensive, and inefficient in terms of cost for real-time implementation. In this review, we first discuss the current state-of-the-art AI models utilized for ECG-based cardiac rhythm classification. Next, we present some of the upcoming modeling methodologies which have the potential to perform real-time implementation of AI-based heart rhythm diagnosis. These models hold significant promise in being lightweight and computationally efficient without compromising the accuracy. Contemporary models predominantly utilize 12-lead ECG for cardiac rhythm classification and cardiovascular status prediction, increasing the computational burden and making real-time implementation challenging. We also summarize research studies evaluating the potential of efficient data setups to reduce the number of ECG leads without affecting classification accuracy. Lastly, we present future perspectives on AI's utility in precision medicine by providing opportunities for accurate prediction and diagnostics of cardiovascular status in patients.

## 1 Introduction

Sudden cardiac death (SCD) often manifests as the first symptom of cardiac arrhythmias, leading to an estimated 350,000 fatalities per year in Europe alone [1]. An abnormal cardiac rhythm, arrhythmia, can be instigated by conduction irregularities originating from heterogeneities in the regional electrophysiologic properties (refractory period, voltage and conduction velocity) of the cardiac tissue [2], [3]. About 1.5-5% of the general population is purported to manifest either atrial or ventricular arrhythmias [4], which are associated with substantial morbidity, increased risk of strokes, hospitalizations and mortality, hence creating a substantial economic and healthcare burden [5].

The measurement of voltage variations brought on by the heart's electrical activity over time provides a mathematical explanation for how an electrocardiogram (ECG) works. The electrical impulses the heart produces regulate its contraction and relaxation, causing little variations in voltage on the skin. Electrodes are positioned in precise areas to record these changes in lead systems like the 12-lead ECG. The resulting waveforms, P, QRS, and T, represent the depolarization and repolarization of the atrium and ventricle, respectively, and correspond to distinct phases of the cardiac cycle. The heart's conduction system can be inferred from the time intervals between these waves, and the R-R interval can be used to compute heart rate [6]. ECGs are pre-processed in several processes to improve signal quality and enable reliable interpretation. Specifically, the pre-processing steps include filtering to remove baseline wander and powerline interference, noise reduction via high-pass filtering and artifact removal, amplitude normalization for consistency, R-peak detection to identify ventricular depolarization, segmentation to isolate individual beats, quality control to address artifacts, resampling for a uniform sampling rate, interpolation to handle missing data, and heart rate normalization for comparative analysis [7]. These pre-processing stages ensure that ECG data is cleaned, standardized, and ready for proper interpretation by healthcare experts, hence assisting in diagnosing and monitoring cardiac problems.

The most common phenotypes of ventricular arrhythmias known to instigate SCD are ventricular tachycardia (VT) and ventricular fibrillation (VF). The implantable cardioverter defibrillator (ICD) is the primary and most effective means of treating lethal VT/VF [8]. Over 200,000 ICD implantations are reported every year [9], yet due to the structural complexity of the human heart, effective control of the heart rate and prevention of fatal rhythms has been challenging. Similarly, atrial tachyarrhythmias, even though less life-threatening than VT/VF, constitute a significant health risk with atrial fibrillation (AF) afflicting over 2.3 million people in the US [10]. AF is one of the most significant causes of stroke, and the current antiarrhythmic drug therapy or catheter ablation treatment strategies are only partially effective. Early detection of AF is essential to prevent the associated comorbidities of AF [11] and enable appropriately targeted treatment [12].

**Figure 1:**
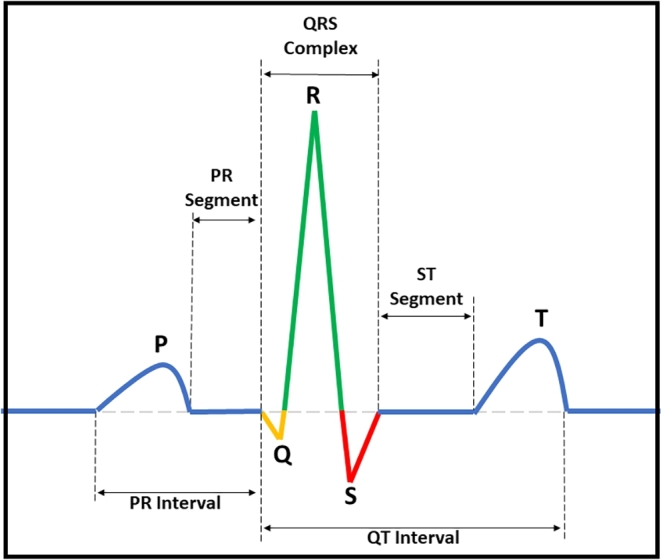
Waveform depicting crucial components of a standard Electrocardiogram (ECG), primarily showcasing atrial depolarization (P-wave), ventricular depolarization (QRS complex wave), and repolarization (T-wave).

**Figure 2:**
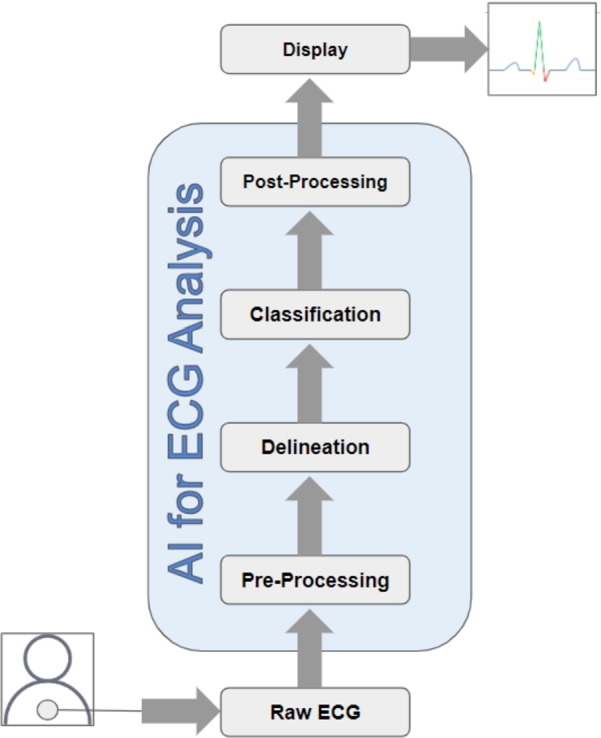
A typical workflow detailing the role of AI in ECG analysis. AI has shown a tremendous performance in pre-processing (denoising), delineation (segmentation of peaks in the ECG signal), classification (of abnormal cardiac rhythms), and post-processing of ECG signals.

The advent of artificial intelligence (AI) and advanced computational techniques has substantially revolutionized healthcare diagnostics [13]. Over the past decade, novel machine learning (ML) and deep learning (DL) modalities have been investigated that utilize ECG data and other vital sign measures to predict cardiovascular status in patients and assist physicians in diagnosis [14], [15], [16], [17], [18], [19], [20], [21], [22], [23], [24], [25], [26]. In particular, there has been a great emphasis on the identification and classification of cardiac rhythm based on the ECG data to aid in prediction of impending arrhythmias and enable timely application of therapy. AI based methods incorporating neural networks, Bayesian networks, fuzzy logic systems, as well as machine learning models using linear or logistic regression, decision trees, k-nearest neighbors, random forest, or support vector machines, have been shown to accurately predict cardiovascular outcomes in patients [27], [28], [14], [16]. Furthermore, the 12-lead ECG based classification algorithms have demonstrated a great precision in early diagnosis of cardiac arrhythmias, highlighting the potential of AI based approaches in supporting cardiovascular patient management [29], [30], [31]. Table 1 lists the details of the commonly identified abnormal cardiac rhythms using AI models. A sample ECG signal with atrial depolarization, ventricular depolarization, and repolarization has been shown in Fig. 1. Further, a typical workflow detailing the role of AI in ECG analysis has been shown in Fig. 2. As shown in Fig. 2, AI has shown a tremendous performance in pre-processing (denoising), delineation (segmentation of peaks in the ECG signal), classification (of abnormal cardiac rhythms), and post-processing of ECG signals.

**Table 1:** Types of abnormal cardiac rhythms, their physiological origins, and definitions.

Cardiac Rhythm	Arrhythmia Origin	Definition
Atrial flutter	Supraventricular	Atrial flutter is a type of abnormal cardiac rhythm that originates in the supraventricular region of the heart, specifically in the atria. It is characterized by an abnormally fast but regular beating of the atria.
Atrial fibrillation	Supraventricular	Atrial fibrillation (AF) is another type of abnormal cardiac rhythm that originates in the supraventricular region of the heart, specifically in the atria. It is characterized by an irregular, rapid, and chaotic beating of the atria.
Sinus arrhythmia	Supraventricular	Sinus arrhythmia refers to a variation in the normal sinus rhythm of the heart, specifically in terms of the timing of the heartbeats causing > 0.12 s change in RR interval
Bradycardia	Supraventricular	Bradycardia is a condition characterized by an abnormally slow heart rate, specifically a resting heart rate of fewer than 60 beats per minute (BPM) in adults.
Premature atrial contraction	Supraventricular	Premature atrial contraction refers to an abnormal heart rhythm characterized by an early, extra heartbeat originating from the atria (the upper chambers of the heart).
Sinus tachycardia	Supraventricular	Sinus tachycardia is a condition characterized by a faster-than-normal heart rate originating from the sinus node, the heart's natural pacemaker located in the right atrium.
1st degree AV block	AV node	In first-degree AV block, there is a delay in the conduction of impulses through the AV node, resulting in a prolonged PR interval on the ECG (> 0.2 s).
Right bundle branch block	His-bundle	Right bundle branch block occurs when there is a delay or blockage of electrical impulses in the right bundle branch, one of the pathways that carries electrical signals to the ventricles.
Left bundle branch block	His-bundle	Left bundle branch block occurs when there is a delay or blockage of electrical impulses in the left bundle branch leading to intraventricular dyssynchrony and delayed activation of the left ventricle.
Premature ventricular contraction	Ventricular	Premature ventricular contraction occurs when an extra electrical impulse originates in the ventricles before the next expected normal heartbeat. This results in interrupting the regular heart rhythm.
Prolonged QT interval	Ventricular	Prolonged QT interval refers to an abnormality in the electrical activity of the heart, specifically in the measurement of the QT interval on an electrocardiogram (ECG). The QT interval represents the time it takes for the ventricles to depolarize and repolarize during each heartbeat.
Ventricular tachycardia	Ventricular	Ventricular tachycardia (VT) is a type of abnormal heart rhythm characterized by a rapid and regular heartbeat originating from the ventricles (the lower chambers of the heart) (> 100 bpm).
Ventricular fibrillation	Ventricular	In Ventricular fibrillation, the ventricles quiver or fibrillate instead of contracting effectively, preventing the heart from pumping blood efficiently.

The contributions listed in refs. [32], [33], [34], [35] provide comprehensive review on ECG based Arrhythmia/Cardiac Rhythm classification. In addition to the comprehensive discussion on AI models for ECG classification, in this review we provide a systematic evaluation/categorization of “Efficient AI” models for ECG based “Cardiac Rhythm” classification. The “Efficient AI” models for ECG classification have been introduced/discussed under three categories (1) Minimum ECG Lead Input Models (cost efficient), (2) Lightweight Deep Learning Models (computation efficient) and, (3) Data Annotation Efficient Models (cost and time efficient). The rest of the manuscript is organized as follows: Section 2 details various databases used to identify the recent developments in ECG classification tasks. Section 3 discusses the relationship between artificial intelligence, machine learning, and deep learning. A detailed analysis of AI models for ECG classification has been discussed in section 4. Section 5 provides a discussion on efficient AI methods for ECG classification. Finally, future perspectives on the role of AI in precision medicine and telehealth have been provided in section 6.

## 2 Search strategy

The two major databases, Google Scholar and PubMed were comprehensively searched without language restriction. The literature search focused on novel ML and DL techniques for ECG classification using 12-lead signals and optimized lightweight models, predominantly developed within the past five years. Particular emphasis was laid on the methodologies developed as a part of the Physionet 2020 and 2021 challenges. The following keywords were used as search criteria: “(deep learning OR artificial intelligence OR machine learning OR prediction of cardiac arrhythmias OR lightweight models OR Physionet challenge) AND ECG classification.”). Before presenting a comprehensive review, we present a generic overview of AI, ML, and DL in the following section.

## 3 Artificial intelligence

Artificial intelligence has been described as the science and engineering of making intelligent machines [37] by John McCarthy. In recent years, its essence has encompassed making models using computer to make cognitive decisions for real-life problems without or minimal involvement of human. Over the past two decades, it has influenced several domains of applications, varying from visual perception to making intelligent robots to knowledge representation, automatic programming, and automatic reasoning [38]. AI-based analytics is gaining popularity in medical diagnostics as it can analyze large magnitudes of complex patient data and exploit the relationships within a dataset to treat and predict diseases [39]. Machine learning is a subset of AI, continuously evolving and utilizing computational algorithms for emulating human intelligence by enabling adaptive learning from the surrounding environment [40]. At the core of ML lies determination of patterns in the data which can eventually be used for analyzing unseen situations. DL is further subset of AI and ML adhering to same objectives as ML but it primarily comprises of multi-layers of neural networks. The presence of several hidden layers (hence the terminology as Deep Learning) make them more efficient and effective over a wider range of data and applications. Lately, it has been extensively used in the area of healthcare such as medical imaging as well as healthcare data analysis for various tasks, including classification [41], [42], segmentation [43], [44], as well as reconstruction [45], [46], [47].

**Figure 3:**
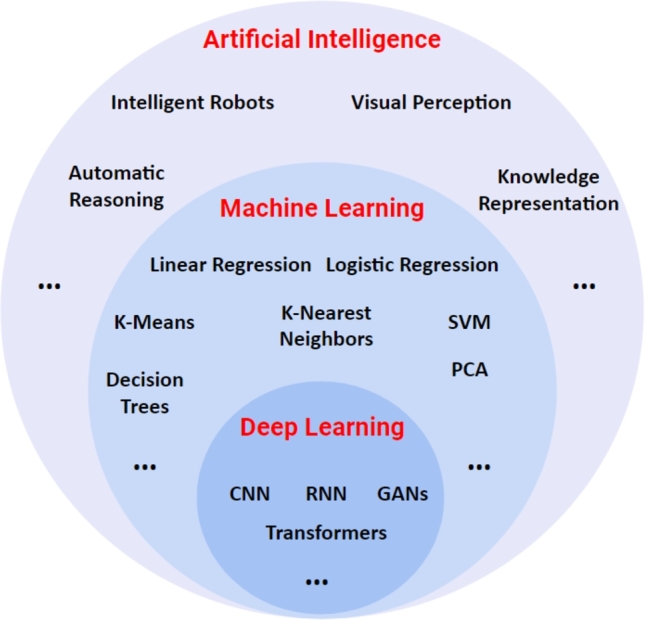
Relationship between artificial intelligence, machine learning and deep learning. SVM : Support Vector Machines; PCA : Principal Component Analysis; CNN: convolutional neural network; RNN: recurrent neural network; GAN: generative adversarial network. (The figure has been adapted and modified from [36]).

**Figure 4:**
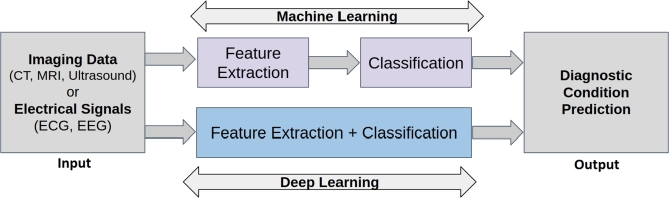
Overview of machine learning and deep learning models for a sample medical data classification task.

In the past five years, immense progress in DL algorithms has been achieved, with many research groups actively investigating novel DL models with enhanced capabilities for healthcare diagnostics [48], [49], [50]. The relationship between AI, ML, and DL is demonstrated in Fig. 3. The fundamental difference between ML and DL is shown in Fig. 4. ML involves manually choosing important information (feature extraction) from the data and using them to make predictions. On the other hand, DL uses artificial neural networks (or its variants) to automatically learn essential patterns from the raw data. DL needs a lot of data to learn well and requires powerful computers. ML is more interpretable because humans choose/manually craft the important information, while DL is often more accurate but harder to understand. As illustrated in Fig. 4, for a sample classification task (predicting diagnostic condition: benign or malignant) on medical data, ML algorithms need to craft features manually for performing the classification whereas the DL models automate the features extraction and classification task. In the following sub-sections, we discuss ML and DL models relevant to the current review in some detail which will facilitate better understanding of utility of these methodologies for the problem in hand.

### 3.1 Machine learning

ML methods are greatly influenced by handcrafted feature representations. For example, in the context of ECG signal processing, these features encompass time-domain characteristics like mean, standard deviation, and heart rate variability measures [51], frequency-domain components such as power spectral density, statistical measures including skewness and entropy [51], and wavelet-based features [52] capturing localized changes and frequency patterns. These ML features play a crucial role in analyzing and classifying ECG data for arrhythmia detection and disease diagnosis, aiding in interpreting ECG signals.

### 3.2 Deep learning

The term “Deep learning (DL)” was coined by Hinton et al. [53] and was mainly built upon the concept of artificial neural networks (ANNs). As a branch, DL falls under a giant bucket of AI and ML (Fig. 3). It involves building various neural networks so that these models mimic how the human brain processes the data. These models have found application in multiple fields, such as smart healthcare, robotics, natural language processing (NLP), cyber security, etc. ANNs constitute multiple layers, which are abstract representations of data being used while training them. Training these ANNs often involves a massive chunk of data (hence sometimes called “data hungry” models). However, once trained, these models provide high accuracy and low inference time compared to other ML algorithms [54]. Here, we present an overview of some commonly used DL architectures like Convolutional Neural Networks (CNNs) and Recurrent Neural Networks (RNNs) for ECG processing.

#### 3.2.1 Convolutional neural networks

Convolutional Neural Networks (CNNs or ConvNets) are popular DL architectures that have found applications in almost all fields ranging from medical image analysis, visual recognition, NLP, and many more such applications. A commonly used CNN consists of multiple convolutional layers followed by sub-sampling (pooling) layers while ending layers vary with the task at hand; for example, the last layers may be fully connected layers for classification tasks and convolutional layer for tasks like segmentation and super-resolution. The key benefits of these networks are equivalent representation, sparse interactions, and parameter sharing [55]. The convolution operation involved in various convolutional layers has minimal parameters, which helps in quick training and avoiding overfitting. Currently, in the literature, there exist several variants of CNNs like AlexNet [56], VGG-Net [57], ResNet [58], etc., that can be used for various tasks at hand according to their learning capabilities [55].

#### 3.2.2 Recurrent neural networks

A recurrent neural network (RNN) is another famous DL architecture that has found its applicability in time-series data processing [59], [60], [61]. Major applications are in speech processing [62], NLP [63] and sequential medical data like EEG [64], ECG [65] etc. Compared to standard CNN architecture, RNN architecture has “memory”, which allows them to predict the current output using the present and previous inputs the network has already seen. Such architecture helps decode vital information embedded in the data sequence. For example, RNNs in the context of ECG can be used to understand the temporal dependencies between successive heartbeats [66].

Keeping aside the advantages that the RNNs offer, the standard RNN architecture has the severe issue of vanishing gradients [67], making learning long sequences in the data quite challenging. Long short-term memory (LSTM) [68] architecture was designed to address this issue. LSTM architecture introduces a particular block called a “Memory Cell”, which can store data for extended periods without forgetting, making learning more efficient. Three gates control the flow of information in and out of this block, first, the “Input Gate”, which determines which information should enter the cell; second, the “Output Gate”, which determines and controls the current cell output; and finally the “Forget Gate” which is responsible for deciding which information from the previous state cell should be memorized and which data should be removed and forgotten. LSTM is considered one of the most successful RNN architectures and has found its application in many real-world domains.

Other popular RNN architectures include bidirectional LSTM/RNNs and gated recurrent units (GRUs) [69], similar to normal LSTM but with subtle differences. For instance, BiLSTM comprises two standard LSTM blocks: one takes input forward, and the other takes it backward, allowing them to access data from both the future and the past. GRUs are similar to LSTM but with only two gates: the “Reset Gate” and the “Update Gate”. Compared to LSTMs, GRUs are quite computationally fast and can capture significant sequence dependencies without discarding information [70].

## 4 AI models for ECG cardiac rhythm classification

### 4.1 CNN based models

Many models using DL-based approaches have been proposed for the classification of ECG using traditional 12-lead signals [71]. In the PhysioNet 2020 challenge, various novel algorithms were proposed for classification using an extensive database comprising 66,361 12-lead ECG recordings. This section presents an overview of some of these novel classification techniques. An algorithm using a transformer-based neural network scored the highest among 41 participating teams [72], which utilized a generalized weighted accuracy metric for evaluation. Their model combined ECG features, extracted by an ML random forest model, and discriminative feature representations automatically learned from a transformer neural network providing an increased classification accuracy. Another group presented a modified ResNet model that used a large kernel size to train long-term dependencies with a squeeze and excitation layer (SE) embedded into the modified ResNet [73] that proved highly effective in 12-lead ECG classification. A similar SE-ResNet based approach incorporated a rule-based model and a “sign loss” to tackle class imbalance, improving the model's generalizability and the classification performance [74]. In another novel implementation, [75], the attention mechanism was combined with a dual-channel deep neural network, which enabled the network to capture richer arrhythmia information leading to efficient ECG classification. Several other approaches have been proposed using different models to enhance the performance of the 12-lead classification algorithms [76].

Garcia et al. [77] integrated a deep branch composed of a modified ResNet with dilated convolutional layers and squeeze and excitation block, concatenated with a wide branch that integrated 20 cardiac rhythm features into a fully connected 3-layered network. Similarly, a model based on the ResNet architecture with a multi-head attention mechanism demonstrated that the multi-head attention layer might not significantly impact the final classification performance [78]. Five models based on deep residual convolutional neural networks were optimized using an asymmetric loss function to classify multi-lead ECGs. More recently, lead-wise relations were captured in a study [79] using a SE deep residual network. The authors proposed a cross-relabeling strategy and applied the sign-augmented loss function to tackle the corrupted labels in the data. Furthermore, they utilized a pos-if-any-pos ensemble strategy to handle the uncertainty of the data distribution in the application. To provide a more detailed description of the issues associated with label mapping, a new model ResNet50 was developed [80] to obtain a better starting point for training more powerful classification models. Kang et al. [81] developed a ‘lead-wise’ mechanism to facilitate parameter reuse in models. This mechanism reduces model sizes while keeping comparable performances. Similarly, another [82] deep residual inception network was recently proposed with a channel attention mechanism that facilitates efficient computation and prevents overfitting while exploring deeper networks through dimensionality reduction and stacked 1-dimensional convolutions.

Different electrophysiological features have been extracted from 12-lead ECG data to identify cardiac arrhythmias with varying classification accuracy. Jimenez et al. [83] extracted 81 features per ECG-lead based on heart rate variability, QRST patterns, and spectral domain and applied one-versus-rest classification from independent binary classifiers for each cardiac condition. A classification model among two binary supervised classifiers and one hybrid unsupervised-supervised classification system was then selected for each class. Wickramasinghe et al. [84] preprocessed recordings shorter than 20s by normalizing, resampling, and zero-padding. The frequency domains of the recordings were obtained by applying a fast fourier transform. The time domain and frequency domain of the signals were then fed into two separate deep convolutional neural networks. The outputs of these networks were then concatenated and passed through a fully connected layer. Outputs were interpreted using Shapley additive explanations for the 2-lead model, which provides insight into labeling inconsistencies and reasons for the model's poor performance. A voting-based lead-agnostic hybrid classifier that combined features extracted through a CNN with hand-crafted features was recently developed [85]. The method improved some individual class classifications but did not offer better generalization than the baseline DL approach. Zhao et al. [86] proposed a SE-ResNet to automatically learn deep features, augmented with age and gender features in the final fully connected layers. They also used two expert clinicians independently to interpret a random set of 100 misclassified ECGs concerning left axis deviation and discovered a considerable inconsistency in training labels, likely hindering the development of more accurate models. In another study, it was shown [74] that an ensemble of two SE-ResNet models and one rule-based model combined with a Sign Loss to tackle the problem of class imbalance improved the model's generalizability.

### 4.2 RNN based models

An LSTM based ensemble classification model resulting from two different feature sets wherein the first feature set extracts RR interval variability by deploying Fourier-Bessel expansion and the second feature set comprises of time- and frequency-domain-based hand-crafted features was recently developed [87]. Another classifier comprised of four modules: scattering transform (ST), phase harmonic correlation (PHC), depthwise separable convolutions (DSC), and LSTM network was developed for cardiac rhythm classification [88]. The ST captures short-term temporal ECG modulations, while the PHC characterizes the phase dependence of coherent ECG components. The output is provided to DSC, which combines lead responses separately for each ST or PHC coefficient and then combines resulting values across all coefficients. The two LSTM layers integrate local variations of the input over long-time scales. Lastly, canonical correlation analysis enables transfer learning from 12-lead ST and PHC representations to reduced-lead ones. In a comparative study, Puszkarski and colleagues [89] analyzed four architectures (N-BEATS, LSTM, LSTM with peepholes, GRU) for different numbers of leads (2, 3, 4, 6, 12) and demonstrated that LSTM, GRU, and N-BEATS best followed the performance of LSTM with peepholes.

## 5 Efficient models for ECG cardiac rhythm classification

**Figure 5:**
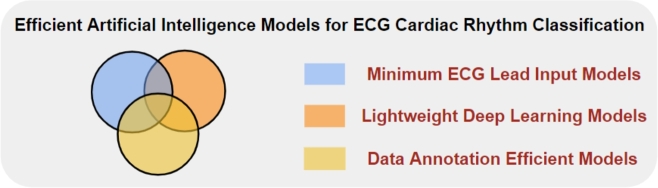
An overview of efficient AI models for ECG cardiac rhythm classification.

Advances in wearable and ambulatory remote monitoring technology [13], [90], [91] have highlighted the need and opportunity to develop AI models which can perform real-time prediction of impending cardiovascular events. While several recent studies have investigated the use of AI in predicting the progression of cardiac diseases for early detection of outcomes using wearable monitoring technology [16], [27], [92], [93], [94], [95], [96], the clinical utility of these models remains limited. Such devices need to be equipped with AI models that are optimized, lightweight, and capable of real-time data processing. This calls for developing optimized AI models that can perform feature extraction using 1 or 2-lead ECG and would be computationally inexpensive. The following subsections discuss the efficient AI models for ECG classification under two categories (Fig. 5): (1) Minimum ECG Lead Input Models, (2) Lightweight Deep Learning Models and, (3) Data Annotation Efficient Models.

### 5.1 Minimum ECG lead input models

The examination of ECGs involves employing both waveform analysis and interval/rhythm analysis. Interval or rhythm analysis focuses on the timing and duration of specific ECG components, such as PR and QT intervals, aiding in the identification of anomalies in heart rhythm. This analysis is crucial for measuring intervals and detecting irregularities. On the other hand, waveform analysis is a crucial diagnostic tool for conditions like myocardial infarction and conduction system disorders, as it scrutinizes the amplitude and shape of the ECG signal. Both approaches utilize algorithms for feature detection and analysis, contributing to a comprehensive understanding of heart function. Integrating these methodologies is essential for clinical ECG interpretation, enabling healthcare professionals to assess each patient's overall heart health thoroughly. Recently, there has been a focused effort to improve the classification of signals by incorporating features with ECG signals through a hybrid approach [97], [98]. Deep learning models are predominantly considered black-box systems, making it challenging to conclusively determine whether these models rely solely on interval measurements or also take into account interval dispersion, potentially resulting in similar performance outcomes. Various investigations have explored the use of gradcam [99], [100] and dual attention [21] based approaches to pinpoint specific segments of ECGs that play a crucial role in classifying diverse ECG signals.

In order to develop optimized models based on minimal inputs and ECG leads, feature engineering plays an important role. The lower dimensionality of information due to the reduced number of ECG leads needs to be mapped with higher dimensionality data from the standard 12-leads. Mapping these two could bridge the gap and improve model prediction, enabling their application in wearable devices and telehealth platforms. A summary of such minimum lead AI models for ECG cardiac arrhythmia classification has been shown in Table 2. Automatic feature extraction from raw data to uncover the underlying characteristics, which eventually facilitate classification, is an essential characteristic of DL models. The strength of DL models lies in their ability to exploit the property as many natural signals are compositional hierarchies, enabling the extrapolation of a high-level feature utilizing a low-level feature. Recently, Linschmann et al. [101] developed a deep neural network for classifying cardiac rhythm abnormalities using ECG data as part of the PhysioNet/Computing in Cardiology Challenge 2021, which consisted of 131,155 12-lead ECG recordings of different lengths and frequencies, labeled with one or more of distinct 133 classes. Their architecture comprised a CNN, a parallel LSTM, a linear network structure for extracting the features, and a sigmoid activation layer that enabled a multi-label classification. Interestingly, their classification model maintained the same efficiency for classification even as the number of leads utilized was reduced from 12 leads to 2 leads [101]. Another group that participated in the same challenge demonstrated superior classification scores of 0.48, 0.48, 0.47, 0.47, and 0.45 out of 1 for the 12-lead, 6-lead, 4-lead, 3-lead, and 2-lead versions of the hidden test set based on the Challenge evaluation metric [102]. Their model utilized EfficientNet-B3 [110] in conjunction with data augmentation, label masking dealing with multiple data sources, threshold optimization, and feature extraction, yielding better classification performance. This can be particularly useful for applications in wearable devices where the small architecture of the device may not permit a high computational setup to be built in, and prediction would be needed to be made using minimal ECG leads.

**Table 2:** Summary of minimum lead input AI models for ECG cardiac arrhythmia classification.

Study	Method/Model	Dataset	Key Contributions
[101] (2021)	CNN+LSTM	PhysioNet-2021	A hybrid CNN+LSTM model with a linear network structure for feature extraction enabling robust multi-label classification.
[102] (2021)	EfficientNet-B3	PhysioNet-2021	A label masking strategy along with threshold optimization for final model predictions enabling superior performance.
[103] (2021)	SE-Net	PhysioNet-2021	A channel wise self attention module within a squeeze and excitation (SE) network for improved classification accuracy.
[104] (2021)	Transformer	PhysioNet-2021	A weighting mechanism using attention modules enabling the focus of neural network onto the relevant features of the input.
[105] (2021)	ResNet	PhysioNet-2021	A ResNet deep neural network architecture with a multi-head attention mechanism foe accurate ECG classification.
[106] (2022)	2D-CNN	PhysioNet-2021	A multi-scale deep neural network with weighted focal loss for ECG arrhythmias classification on varying dimensional inputs.
[107] (2021)	Boosting	PhysioNet-2021	A mel-frequency cepstrum and amplitude-time heart variability features, handcrafted for ECG arrhythmia classification.
[108] (2021)	SqueezeNet	PhysioNet-2021	A wavelet transform coefficients based scalogram features for ECG arrhythmia classification using deep neural networks.
[109] (2021)	SE-ResNet	PhysioNet-2021	A self-supervised auxiliary task for peak detection on signals with different sampling rates enabling robust ECG classification.

Among several other available models, channel self-attention-based deep neural networks have been proposed to diagnose various cardiac abnormalities, not only with 12-leads but even with fewer ECG leads [103]. The strength of this approach lies in facilitating the inspection and categorization of inter-beat and intra-beat patterns. The model works by squeezing the global spatial information and generating a channel-wise statistic using the channel self-attestation-based framework. Classification accuracy was improved by assigning a higher weight to the more imperative channel resulting in enhanced performance [103]. Another model that has shown immense potential is the waveform transformer, which weighs different parts of the input using an attention mechanism. The attention maps from this model further provide insight into the parts of input data that play a significant role in the final prediction [104]. Nejedly et al. [105] reported a ResNet deep neural network architecture with a multi-head attention mechanism that accurately performs ECG classification with equal efficiency using a variable number of leads ranging from 12-leads, 6-leads, 4-leads, 3-leads to 2-leads. Their model was optimized using a mixture of loss functions, binary cross-entropy, custom challenge score loss function, and sparsity loss function, and probability thresholds were estimated using the evolutionary optimization method [105].

Xia et al. [106] recently proposed a novel multi-scale 2D CNN that can accurately identify 30 arrhythmias from 12-lead, 6-lead, 4-lead, 3-lead, and 2-lead ECG signals. Furthermore, they demonstrated that reduced-lead models could achieve comparable classification performance to the standard 12-lead model. Other models, such as the XGBoost that utilize mel-spectrograms and heart rate variability as features [107], and a SqueezeNet-based DL model that uses continuous wavelet transform coefficients [108] for ECG classification, have also been reported, albeit with slightly lesser efficiency. Li et al. developed a model to classify abnormalities using reduced-lead ECG recordings based on SE-ResNet [109]. Their methodology consisted of peak detection as a self-supervised auxiliary task and integrating models of different sampling rates and input lengths. Optimal settings were selected based on simultaneous consideration of intra-source performance and inter-source generalization, using which the authors achieved consistent classification accuracy for multiple lead combinations of 12-leads, 6-leads, 4-leads, 3-leads, and 2-leads.

### 5.2 Lightweight deep learning models

**Figure 6:**
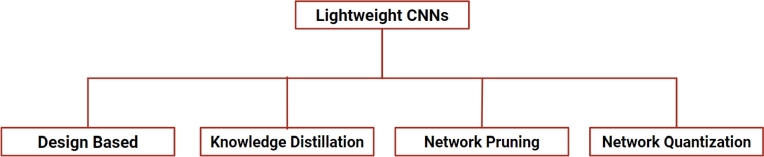
Different techniques used for designing lightweight CNNs.

Lightweight CNNs are critical because of the growing demand for efficient and resource-constrained applications. They solve computational resource constraints, like processing power and memory, by providing computationally efficient structures. They are helpful for time-sensitive applications due to their faster inference times, which enable real-time and low-latency processing. In the rapidly evolving landscape of medical research and technology, the continuous updating of DL models is indispensable. This involves integrating new data, adapting to emerging patterns, and enhancing overall model performance. In the context of ECG analysis, DL models can be consistently adjusted to new cardiac conditions, incorporate data from ongoing clinical trials, and accommodate changes in patient demographics. For instance, advancements in DL models for arrhythmia detection may aim to cut down training time from several weeks to just a few hours, facilitating regular updates and implementation. Initiating these updates is particularly feasible in lightweight models, positioned at the forefront of enhancing adaptability and efficiency within the overall system. A summary of such lightweight AI models for ECG cardiac arrhythmia classification has been shown in Table 3. Lightweight CNNs are specifically designed to function on resource-constrained devices like mobile phones, wearables, and IoT devices, where memory footprint and computational requirements must be kept to a minimum. The main techniques for implementing lightweight models can be broadly categorized into four types: (a) Design Based Architectures, (b) Knowledge Distillation, (c) Network Quantization, and (d) Network Pruning (Fig. 6). This sub-section introduces the abovementioned techniques and discusses the contemporary lightweight networks used for ECG classification.

**Table 3:** Summary of lightweight deep learning models for ECG cardiac arrhythmia classification.

Study	Method/Model	Dataset	Key Contributions
[118] (2020)	PD-Net	MIT-BIH arrhythmia database	A lightweight network employing point-wise and depth-wise convolution operation for detecting different types of heart arrhythmias.
[119] (2018)	LiteNet	MIT-BIH arrhythmia database	A efficient network capable of getting trained on low-capacity servers and being deployed on resource-constrained mobile devices for arrhythmia detection.
[120] (2022)	ResNet/MobileNets	Proprietary	A comprehensive study demonstrating efficient deployability of MobileNets over ResNets on embedded wearable devices.
[121] (2022)	CNN	Chapman ECG database	A knowledge distillation technique for constructing efficient single lead network (Student Model) from multi-lead network (Teacher model)
[122] (2020)	CNN	PhysioNet/CinC - 2017	A knowledge distillation approach was utilized to bridge the performance drop due to binarization (for model deployability) of a convolutional neural network.
[123] (2023)	CNN	PTB-XL, CPSC 2018, HFHC Datasets	A knowledge distillation technique was used to distill the performance of the six-view networks (Teacher Network) into a single-view network (Student Network).
[124] (2023)	MVKT-ECG	PTB-XL, ICBEB 2018	A knowledge distillation framework to distill the knowledge of the superior model from multiple views of ECG (e.g., 12-lead ECG) to single-lead-based ECG
[125] (2023)	1D-CNN	PhysioNet-2017	A pruning strategy was employed on 1D-CNN architecture for arrhythmia classification. Trade-off analysis was carried out between accuracy and computational complexity.
[126] (2023)	LightX3ECG	Chapman ECG, CPSC 2018	Advanced pruning strategies were explored to build efficient models in terms of accuracy (F1-score), compactness, and complexity.
[127] (2023)	CNN	MIT-BIH arrhythmia database	A tiny matched filter-based CNN was developed for arrhythmia classification. Various state-of-the-art pruning methods were employed to enable deployment on edge devices.
[128] (2022)	1D-CNN	MIT-BIH arrhythmia database	A feasibility study was conducted to demonstrate a post-training 8-bit integer quantization technique for ECG arrhythmia classification.
[129] (2020)	CNN	MIT-BIH arrhythmia database	A quantized CNN network was proposed to aid the real-time deployment of the arrhythmia detection system on the edge device.

#### 5.2.1 Design based architectures

Architectural design-based lightweight models have performed on par with existing state-of-the-art heavy models. MobileNets [111] utilize depth-wise separable convolutions [112] for reducing the effective number of parameters in a given convolution layer. Recently. Sandler et al. [113] proposed a variant of MobileNet called MobileNetV2 with inverted residual blocks for efficient feature propagation. Another variant of this model, MobileNetV3 [114], utilizes a hardware-aware neural architectural search for developing next-generation MobileNets. Zhang et al. introduced ShuffleNets [115] that utilize channel shuffle operations and pointwise group convolutions for efficient feature propagation. ShuffleNetV2 [116] considers several practical guidelines (channel shuffle, pointwise group convolutions, width multiplier for controlling model complexity) for designing extremely lightweight models. The family of EfficientNets [110] [117] have systematically studied the scaling of deep models across the depth, width, and spatial extent of feature maps. Such a scaling mechanism helps in improving accuracy and resource utilization compared to MobileNets.

In the effort to achieve real-time deployment of various ECG systems, a noticeable transition has occurred, moving away from complex deep neural networks (DNNs) towards designing more lightweight DNNs. A lightweight network called PD-Net [118] was designed to employ a point-wise convolution layer clubbed with a depth-wise convolutional network for arrhythmia diagnosis. Another network called LiteNet [119] was designed to balance the trade-off between model complexity and model accuracy. The LiteNet model was trained on low-capacity servers and was deployed over resource-constrained mobile devices for arrhythmia detection with a low memory footprint. Results of both PD-Net & LiteNet were demonstrated on MIT-BIH ECG databases. More recently, another study [120] was conducted in 2022 to evaluate compressed deep learning models for classifying arrhythmia over embedded wearable devices. The results of the study [120] demonstrated that MobileNets should be preferred over ResNet based models.

#### 5.2.2 Knowledge distillation

Knowledge distillation [130] is a prominent ML strategy for transferring knowledge from a large, complex model (the teacher) to a smaller, more efficient model (the student). The student model is trained to mimic the output of the teacher model, often by minimizing the difference between their predictions. The literature on knowledge distillation spans a wide range of topics, including distillation methods such as attention-based approaches and ensemble-based techniques [131], as well as applications in fields such as computer vision and NLP.

Majid et al. [121] introduced a knowledge distillation based approach to bridge the gap between the high-performing arrhythmia classification model based on multi-lead ECG signals and its counterpart using single-lead ECG signals. The proposed framework comprises a sophisticated teacher model built on multi-lead ECG signals and a simplified student model designed for single-lead ECG signals. Authors [121] report 262.18 times compression compared to the actual teacher model with only 0.81% loss in accuracy. The performance was evaluated on the Chapman ECG dataset [132]. Another work [122] performed binarization of a convolutional network to deploy the model in computing-resource-constrained environments. They used a knowledge distillation approach to compensate for the performance dip due to binarization. The effectiveness of this approach was demonstrated on the PhysioNet/CinC AF Classification Challenge 2017 dataset, where the F1 score decreased slightly from 0.88 to 0.87. The authors in [123] tackle the challenging task of multi-label classification by adopting a multi-view learning approach. Their strategy not only facilitates them to extract ECG features of high quality but also helps them capture distinct features from different ECG leads. Since their model used six view networks, it posed various computation-related deployment bottlenecks. To address this, they employed a knowledge distillation technique, which helped them distill the performance of the initial six view networks into a single-view network. Interestingly, the student network gave superior performance to the teacher network. The performance was demonstrated on three publically available dataset namely: PTB-XL dataset [133], CPSC 2018 Dataset [134] and Hefei High-Tech Cup (HFHC) Dataset [135]. In a similar line, another multi-view technique called MultiView Knowledge Transferring of ECG (MVKT-ECG) [124] was proposed to boost single-lead ECG's ability for multi-label disease diagnosis. They demonstrated using the knowledge distillation technique to distill the knowledge of the superior model from multiple views of ECG (e.g., 12-lead ECG) to single-lead-based ECG. The results were demonstrated on PTB-XL dataset [133] & ICBEB2018 dataset [134].

#### 5.2.3 Network pruning

Network pruning aims at removing redundant channels and kernels to speed up the deep networks during inference [136], [137], [138]. The existing strategies for network pruning involve both; training from scratch with sparsity constraints [139], [140] and compressing an existing pre-trained heavy model using feature level reconstruction errors [141]. Pruning methods are helpful when we move deep learning networks to hardware, facilitating the real-time deployment of computationally demanding models. These techniques are vital in maintaining accuracy and performance when these models are implemented on edge devices. Along these lines, there have been works to construct real-time ECG classification systems. The authors in [125] demonstrate the pruning analysis for the 1D-CNN architecture for arrhythmia classification. When deployed on FPGA, they present various trade-off analyses between accuracy and computational complexity. Another model called LightX3ECG [126] was developed using advanced pruning strategies. Authors of the study [126] claim that their model outperforms most efficient models in terms of F1 score, compactness, and complexity. Recently a tiny matched filter-based CNN [127] was developed for arrhythmia classification. In this work [127], various state-of-the-art pruning methods were employed to enable edge deployment of matched filter-based CNN for arrhythmia classification.

#### 5.2.4 Network quantization

Accelerating deep neural networks and reducing memory footprint is crucial for designing efficient networks. Network quantization aims at converting floating-point parameters (and operations) to low-precision fixed integer types, enabling reduced memory usage with an accelerated inference [142]. Similar to network pruning methods described in the previous section, network quantization techniques help in the real-time deployment of the deep learning model for various tasks at hand. The significant advantage these techniques offer is fast inference time on the hardware. Recently a feasibility study [128] was conducted to demonstrate a post-training 8-bit integer quantization technique on deep convolutional neural network for automated classification of five different arrhythmia categories. The final efficient model had a lower memory footprint and latency than the non-quantized model. Another quantized CNN network [129] was proposed to aid the real-time deployment of the arrhythmia detection system on the edge device. The proposed network achieved 58.8x faster inference time than the other state-of-the-art methods.

### 5.3 Data annotation efficient models

The studies above develop and adapt neural network architectures that efficiently identify various rhythm classes. These networks are generally trained under supervision using labeled training data. Another line of ongoing research is to combat the issue of labeled data insufficiency. Many recent studies have adapted the concept of contrastive learning to tackle the challenge posed by unlabeled training data. A summary of such data annotation efficient models for ECG cardiac arrhythmia classification has been presented in Table 4. While most of the studies in contrastive learning [143], [144], [145], [146] have focused on proposing efficient transformation as it has considerable potential to improve the efficacy of existing approaches, some have developed novel patient-specific contrastive loss functions [143] to boost classification accuracy. Kiyasseh et al. [143] surpass the well-known contrastive learning framework SimCLR by 15.8% in terms of area under the curve (AUC) on Chapman dataset [132] by introducing a patient-specific noise contrastive loss and three transformations namely: Contrastive Multi-segment Coding (CMSC), Contrastive Multi-lead Coding (CMLC) & Contrastive Multi-segment Multi-lead Coding (CMSMLC). Another state-of-the-art (SOTA) method self-supervised contrastive learning approach called sCL-ST [147] for multi-label classification of 12-lead ECGs introduced two novel transformations - split-join and semantic weighted peak noise smoothing, which enabled a robust method insensitive to changes in ECG signal. This proposed network achieved SOTA performance on 12-lead PhysioNet 2020 dataset. A few shot learning approach using siamese convolutional networks (SCNN) [148] was recently proposed; this method achieved an accuracy of up to 95% on the publically available INCART 12-lead Arrhythmia dataset [149]. Vázquez et al. adapted a self-learning multi-class label correction method to learn a multi-label classifier for ECG signals and evaluated the model using 5-fold cross-validation [150]. They successfully demonstrated that self-learning label correction can effectively address unknown label noise and improve classification accuracy even with the reduced number of ECG leads.

**Table 4:** Summary of data efficient AI models for ECG cardiac arrhythmia classification.

Study	Method/Model	Dataset	Key Contributions
[143] (2021)	CLOCS	PhysioNet 2020, 2017, Chapman, Cardiology Dataset	A family of patient-specific contrastive learning methods for ECG classification. They also introduce three transformations namely: Contrastive Multi-segment Coding (CMSC), Contrastive Multi-lead Coding (CMLC) & Contrastive Multi-segment Multi-lead Coding(CMSMLC) for aiding self supervised training.
[144] (2022)	CNN	PhysioNet-2020, Chapman Dataset	A Self-supervised representation learning framework for 12 Lead classification. It also demonstrates the impact of self-supervised pre-training which enhances the classification performance of fine-tuned classifiers against physiological noise.
[145] (2020)	CNN	MIT-BIH Arrhythmia Database	A Subject-Aware contrastive learning framework proposed for biosignals (EEG & ECG signals). Patient specific contrastive loss, and an adversarial training was used promote patient-invariance during the self-supervised learning.
[146] (2023)	CNN	PhysioNet-2021	A siamese framework has been proposed to perform efficient pre-training of the CNN using large database of un-annotated (or weakly annotated) ECG data. After pre-training, the CNN model has been fine-tuned for robust ECG classification.
[147] (2023)	sCL-ST	PhysioNet 2020	A contrastive learning approach for multi-label classification. Two novel transformations - split-join and semantic weighted peak noise smoothing is proposed to make network insensitive to noises present in ECG signal.
[148] (2023)	Siamese-CNN	INCART 12-lead Arrhythmia dataset	A few shot learning approach using siamese convolutional networks for Arrhythmia classification. Contrastive Loss was used for training the model which gave best performance, as the loss function
[150] (2022)	1D-CNN	PhysioNet/CinC 2021	A self-learning multi-class label correction method to learn a multi-label classifier in presence of noisy labels.

## 6 Future perspectives: role of artificial intelligence in precision medicine and telehealth

To understand the value of the AI models we create, it is crucial to know their intended purpose, whether they are meant for determining current clinical condition (diagnosis) or predicting future diseases (prognosis) [151]. Additionally, we need to be mindful of the specific group of people for whom the algorithm is designed. For example, there are over 360 models for heart diseases in the general population [152], more than 160 models tailored specifically for women and their heart health [153], and over 80 models dedicated to predicting sudden cardiac arrest [154]. Once we understand the potential clinical condition, we must know precisely where each AI model should be implemented in the clinical workflow to maximize the model performance [151]. Incorrect deployment of an AI model in the clinical workflow may lead to inaccurate inferences, which can be disastrous in critical situations. Another crucial factor to consider before deployment is the choice of evaluation metrics. Many models assess performance using AUROC (area under receiver operating characteristic) and classification accuracy, which may not be entirely suitable in a clinical setting [151]. These metrics may not offer a comprehensive picture of the performance and utility of an AI prediction model. For instance, instead of directly relying on AUROC as the final metric, it is advisable to explore decision curves [155], [156]. Considering false-positive and false-negative predictions, these curves help determine the threshold at which treatment initiation might be considered. Another factor in developing a new AI model involves evaluating its performance compared to the outcomes achieved by clinical experts. While such studies pose inherent challenges described in, they bring the model closer to deployment in a clinical setting and prove more effective in comparison to typical metrics like AUROC.

The previous sections have highlighted the significance of AI and, in particular, the utility of DL for predicting cardiovascular abnormalities from non-invasive ECG. This has been primarily driven by the potential of DL-based modalities to seek insights from subtle variations and patterns inherent in the ECG, which may otherwise be overlooked due to the limitations of human capacity. With nearly a billion registered ECGs across the globe in an entire year, a mammoth database of information is available to be tapped for assessing the abnormalities related to heart diseases and thereby enabling novel technologies for preventive diagnostics. However, there are important challenges in accessing and analyzing this data. First, the myriad data resources must be available in an organized and uniform format, enabling a global platform for developing and sharing methodological advances. In this regard, significant datasets have been made available (such as data from several countries across three continents in PhysioNet/Computing Challenge 2021), marking the first step in this direction. However, most resources remain untapped due to lack of accessibility. Data forms a critical asset in developing AI models, and hence, one of the essential aspects of unfettered but secured data availability remains a primary challenge.

From computer-assisted 12-lead ECG, several features can be extracted which need to be trained by human intervention. One primary challenge addressed recently is extracting similar features using a single ECG lead. This could form the benchmark for a fair comparison between different types of leads used and assess the optimal lead combination required for the highest precision and prediction accuracy. Furthermore, lead optimization would be critical for real-time applications and predicting impending arrhythmic episodes in patients. Combining these real-time AI models with existing clinical parameters such as CHARGE-AF (Cohorts for Heart and Aging Research in Genomic Epidemiology–Atrial Fibrillation) score would also be advantageous, which could potentially improve prediction efficacy.

Due to the limited information available from a single lead, additional physiological features should complement these models. Hence, added vital signs such as blood pressure, heart rate, and SpO2 [157], [158] could be used as complementary features to improve the model accuracy and performance. Though this may pose an initial challenge owing to the need for additional data curation efforts, these models have the potential to be incorporated into wearable devices that already have an established platform for extracting the required desired signs. Furthermore, implementing lightweight real-time AI models in intensive care units, incorporating data from lab tests, patient history, and vital signs such as temperature, respiratory rate, and blood pressure, can further augment the model capabilities, eventually opening the door to provide personalized precision diagnostics. A vast array of wearable monitoring devices and innovative applications are now available that enable the collection of physiological data [16], [159], [160]. Supplementing these with AI-based prediction models can form the basis for the next-generation patient monitoring technology that enables telehealth assessment and remote precision medicine. AI has transformed ECG analysis into wearable technology, offering real-time cardiovascular health information. With the help of these intelligent wearables with sophisticated algorithms, users may proactively manage their heart health as they continuously monitor and interpret ECG data [161], [162]. Artificial intelligence is seamlessly incorporated into ECG interpretations to improve accuracy and facilitate early identification of anomalies and probable cardiac problems. This cutting-edge wearable and AI combination encourages people to be more proactive with their health, ushering in a new era of individualized and preventative healthcare.

Another important consideration for AI-based models is the ability to detect rare diseases which may be missed as outliers using standard approaches and need specialized features for detection. So far, most of the models that have been trained and tested in various data challenges focus primarily on classifying commonly occurring arrhythmias which are a subset of the abnormal cardiac rhythms that can be observed in patients. The true strength of AI-based diagnostics would be availed and defined by its ability to detect common arrhythmia with obvious ECG phenotypes and rare disorders that manifest as subtle changes in morphology or duration of ECG. An exciting approach in this regard, to further enhance the capabilities of AI models, would be to perform feature extraction using DL models and subsequently use ML models for processing the extracted optimized features. This strategy combines the strengths of both the subsets of AI while necessitating a minimal input feature set. Such an approach could be helpful, especially for applications in wearable devices, as the overall computational resources required would be less, simplifying the model implementation.

## CRediT authorship contribution statement

**Utkarsh Gupta:** Conceptualization, Investigation, Methodology, Validation, Visualization, Writing – original draft, Writing – review & editing. **Naveen Paluru:** Conceptualization, Investigation, Methodology, Validation, Visualization, Writing – original draft, Writing – review & editing. **Deepankar Nankani:** Investigation, Methodology, Writing – original draft. **Kanchan Kulkarni:** Investigation, Methodology, Writing – original draft. **Navchetan Awasthi:** Conceptualization, Investigation, Methodology, Project administration, Supervision, Validation, Visualization, Writing – original draft, Writing – review & editing.

## Declaration of Competing Interest

The authors declare that they have no known competing financial interests or personal relationships that could have appeared to influence the work reported in this paper.
